# A pilot study on the acoustic effects of a pseudo-palatal plate on speech: Implications for articulatory rehabilitation devices

**DOI:** 10.1371/journal.pone.0343657

**Published:** 2026-02-26

**Authors:** Seong Tak Woo, Sungdae Na

**Affiliations:** 1 Department of Electronic Engineering, Dong Seoul University, Seongnam, Republic of Korea; 2 Department of Biomedical Engineering, Kyungpook National University Hospital, Daegu, Republic of Korea; Manipal Academy of Higher Education, INDIA

## Abstract

Intraoral palatal plates used in electropalatography (EPG) and tongue-interface systems are designed to monitor articulatory movement with minimal disruption to speech. However, their presence may subtly influence acoustic characteristics by altering tongue mobility and intraoral airflow. This study examines the acoustic effects of a pseudo-palatal plate during the articulation of consonants and vowels. Speech samples from healthy adults were recorded with and without the plate and analyzed using spectral and phonatory measures, including Mel-frequency cepstral coefficients (MFCCs), second formant (F2) slope, jitter, shimmer, harmonics-to-noise ratio (HNR), and quadrilateral vowel space area (qVSA). MFCC-based correlation coefficients and F2 slopes remained highly consistent across conditions, indicating minimal impact on consonant articulation. Shimmer (−0.72%), HNR (+2.5 dB), and qVSA (−24.1%) exhibited directionally consistent changes, with qVSA trending toward reduction; however, overall variation was limited. Vowel formant structures and consonant production were preserved mainly, suggesting that the palatal plate did not significantly impair vocal function. These findings support the interpretation that observed acoustic shifts are more consistent with filter-level effects than with changes at the glottal source. Although palatal plates may introduce minor acoustic variations, their impact on speech production appears minimal, reinforcing their suitability for therapeutic and assistive use, provided that acoustic considerations are addressed during design and clinical implementation.

## Introduction

The tongue plays a vital role in essential bodily functions, including speech, breathing, and swallowing. Its movements involve complex coordinated sensory-motor actions that are both voluntary and involuntary. Such articulatory patterns offer valuable diagnostic insights into neurological impairments, degenerative conditions, and age-related speech disorders [1,2]. Technologies that monitor tongue movement, such as electropalatography (EPG) and tongue interface systems (e.g., palate-mounted pressure sensors or electromagnetic and inductive tongue-tracking devices), have been developed for clinical assessment and assistive applications. EPG is a widely adopted technique that records the timing and location of tongue-to-palate contact during speech using a custom-fitted palatal plate embedded with multiple electrodes. This method yields detailed spatiotemporal articulatory data and is commonly used to diagnose and treat disorders such as dysarthria and apraxia of speech [3–6]. Similarly, intraoral sensors and tongue interfaces have been explored as assistive technologies for individuals with physical disabilities, enabling fundamental interactions such as computer control or wheelchair navigation through tongue gestures [7,8]. For these systems, whether designed for speech therapy or assistive control, palatal sensors must be minimally invasive, comfortable to wear, and cause minimal disruption to natural articulation.

Several studies have fabricated palatal sensors as personalized devices by molding them to the user’s oral cavity. In EPG applications, palatal plates are typically constructed from biocompatible thermoformed sheets less than 100 µm thick to minimize interference with speech production. Despite these customized designs, wearing such sensors can alter intraoral volume and airflow dynamics, potentially affecting speech output [9,10]. Prior research has examined how speakers adapt their articulation and acoustic patterns in response to artificial palatal perturbations. Thibeault et al. used electromagnetic articulography (EMA) to study the effects of varying palatal thicknesses. They reported notable articulatory adjustments, including changes in tongue positioning and jaw movement, when a thick palate was worn [11]. A brief, targeted training session enabled near-complete recovery of original acoustic patterns, suggesting rapid sensorimotor reorganization. Similarly, McAuliffe et al. employed EPG alongside acoustic and perceptual analyses to investigate short- and mid-term adaptation to a thick palatal plate. They observed immediate changes in tongue-palate contact and corresponding acoustic parameters, with partial recovery occurring within 45 minutes to 3 hours [12]. These findings indicate that while adaptation to palatal perturbations is possible, the degree and rate of recovery vary across speech sounds and speaking conditions. In this context, acoustic features provide objective and quantifiable feedback on speech production and are therefore relevant for speech intelligibility assessment, therapeutic feedback, and sensor design. However, the broader acoustic consequences of such devices during natural speech production remain underexplored.

This study aims to investigate the acoustic effects of a palatal plate and propose an evaluation method based on acoustic parameters to inform sensor design and speech therapy applications. To provide a comprehensive assessment of the plate’s impact on articulation, we analyzed consonant and vowel features using both spectral and phonatory measures. For consonants, we examined Mel-frequency cepstral coefficients (MFCCs) and second formant (F2) slope to assess spectral changes. For vowels, we analyzed jitter, shimmer, harmonics-to-noise ratio (HNR), and quadrilateral vowel space area (qVSA).

## Acoustic analysis method

### A. Consonant analysis based on MFCCs

Because vowels and consonants exhibit distinct spectral patterns shaped by vocal tract configurations, MFCCs are well-suited to capture these differences. They are particularly effective in distinguishing consonants, which often display rapid spectral transitions, transient acoustic features, and high-frequency energy. While vowel sounds are defined by relatively stable formant structures, consonants—especially stops, fricatives, and affricates—possess distinctive spectral characteristics that can be effectively represented using MFCCs. Accordingly, MFCCs have been widely applied in consonant classification, phoneme recognition, and the evaluation of articulatory patterns in speech-related research [13–16].

**Fig 1:**
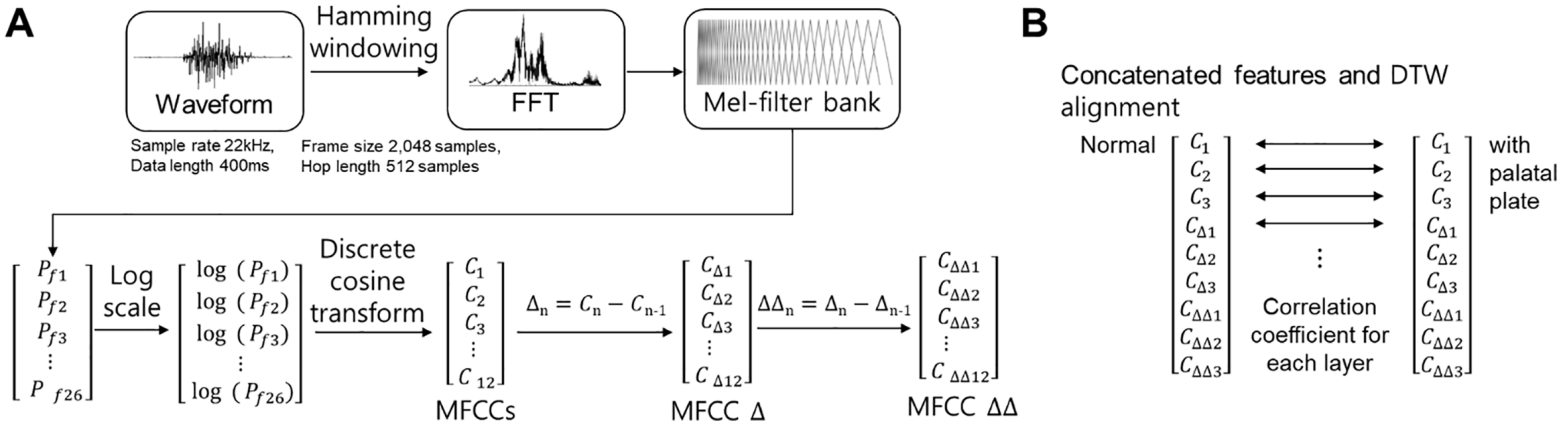
(A) Overview of Mel-frequency cepstral coefficient (MFCC) extraction stages, including waveform segmentation, fast Fourier transform (FFT), and Mel-filter bank processing. (B) Correlation analysis between MFCC layers using Discrete Wavelet Transform (DTW).

In the present study, MFCCs were not treated as novel acoustic descriptors but were used to quantify spectral and temporal changes in consonant articulation induced by the presence of a thin pseudo-palatal plate. The MFCC extraction process is illustrated in **Fig 1(A)**. The resulting MFCCs and their temporal derivatives were used as primary features to analyze articulation characteristics.

MFCC extraction was performed using a sampling rate of 22 kHz. Speech signals were analyzed within a 400 ms analysis segment. Within each segment, overlapping frames of 2,048 samples (approximately 93 ms at 22 kHz) were extracted with a frame shift (hop size) of 512 samples (approximately 23 ms). Each frame was multiplied by a Hamming window to reduce boundary discontinuities prior to spectral analysis. Each windowed frame was transformed from the time domain to the frequency domain using a fast Fourier transform (FFT). The resulting frequency spectrum was passed through a Mel-scaled filter bank consisting of 26 filters, which models the nonlinear frequency resolution of the human auditory system [13,14]. The Mel scale is defined in Equation (1).


$$M(f)=\text{2}\text{5}\text{9}\text{5}\;\times \;{\text{log}}_{\text{1}\text{0}}\left(\text{1}+\frac{f}{\text{7}\text{0}\text{0}}\right)$$ (1)

Where *M*(*f*) denotes the Mel-scaled frequency corresponding to the actual frequency *f*, the filterbank energies are subsequently transformed using a logarithmic function to simulate the human auditory system’s nonlinear perception of loudness. A discrete cosine transform (DCT) is then applied to the log-transformed Mel filterbank energies to generate the MFCCs, which provide a compact representation of the spectral characteristics of the original speech signal. MFCC analyses focused on the first three coefficients (C1–C3), which primarily reflect low-order spectral envelope characteristics associated with vocal-tract configuration. Higher-order MFCCs were not emphasized because they tend to capture fine spectral details and noise-sensitive components that are less directly related to articulatory changes induced by the pseudo-palatal plate. First- and second-order temporal derivatives (ΔMFCC and ΔΔMFCC) were also computed to characterize dynamic articulation patterns. As illustrated in **Fig 1(B)**, DTW was employed to align the time-series patterns of MFCC, ΔMFCC, and ΔΔMFCC features across speech samples, compensating for temporal variations in articulation timing. This alignment enabled robust comparison of phoneme articulation characteristics across different consonant types. The correlation coefficients derived from DTW served as quantitative indicators of similarity between the spectral trajectories of each feature layer, offering deeper insight into the influence of the palatal plate on temporal articulation patterns.

### B. Formants and F2 slope

Formant analysis is a fundamental technique for examining the resonant properties of the vocal tract during speech production. Formants are resonant frequencies shaped by the configuration of the vocal tract and play a central role in defining the acoustic characteristics of speech sounds. The first formant (F1) is generally associated with tongue height, whereas the second formant (F2) reflects tongue advancement [17,18]. In contrast, consonant articulation is more appropriately characterized by dynamic acoustic measures, such as formant transitions and the F2 slope, which capture rapid changes in tongue position during speech. In particular, the F2 slope reflects anterior–posterior tongue movement and has been widely used to describe place-related dynamics in consonant production [19,20]. Prior studies, including those by Tamura et al., have demonstrated that, in individuals with articulation disorders, the F2 slope correlates strongly with reduced tongue movement speed [21]. Specifically, when tongue strength and mobility are diminished, the F2 slope tends to be shallower, indicating slower articulatory transitions. In contrast, no notable changes have been observed in F1 or the third formant (F3) with respect to anterior or posterior tongue positioning. Typically, F1 is more closely linked to tongue height, whereas F3 is associated with pharyngeal constriction [22]. In the present study, we focused on changes in the F2 slope to assess acoustic variation during the production of target consonants while participants wore the palatal plate.

### C. Quadrilateral vowel space area (qVSA)

The palatal plate used in this study, whether for language rehabilitation or as a tongue-to-interface device, may constrain the natural range of tongue movement within the oral cavity. To evaluate such articulatory limitations, the vowel space area (VSA) serves as a relevant acoustic index for assessing articulatory function under plate-wearing conditions. VSA is widely employed in clinical research to correlate pathological speech characteristics—particularly in disorders such as dysarthria and apraxia of speech—with articulatory impairments [23]. In these conditions, vowel centralization commonly occurs due to a reduced range of articulatory motion. VSA is an articulatory–acoustic metric that quantifies the extent of tongue movement within the F1–F2 formant space, presenting the phonetic working space during vowel productions. It is considered a sensitive indicator of articulatory precision and speech intelligibility. In this study, qVSA was calculated using a geometric method based on the F1 and F2 frequencies of four corner vowels:/a/,/i/,/u/, and/e/. These vowels were selected because they represent extreme articulatory positions in terms of tongue height and advancement [24]. Formant values, including the F2 slope, were extracted from the midpoint of each vowel using a formant ceiling of 5000 Hz, a time step of 5 ms, and a window length of 25 ms. The qVSA was computed as the area enclosed by the F1–F2 coordinates of the four vowels, as defined in Equation (2).


$$qVSA\;[Hz^{\text{2}}]=\text{ abs}((F_{{\text{1}}_{i}}\times \left(F_{{\text{2}}_{e}}-\;F_{{\text{2}}_{u}}\right)+F_{{\text{1}}_{e}}\times \left(F_{{\text{2}}_{u}}-\;F_{{\text{2}}_{i}}\right))+F_{{\text{1}}_{u}}\times \left(F_{{\text{2}}_{i}}-\;F_{{\text{2}}_{e}}\right))/\text{2})\;\;+\text{ abs}((F_{{\text{1}}_{e}}\times \left(F_{{\text{2}}_{u}}-\;F_{{\text{2}}_{a}}\right)+F_{{\text{1}}_{u}}\times \left(F_{{\text{2}}_{e}}-\;F_{{\text{2}}_{a}}\right))+F_{{\text{1}}_{a}}\times \left(F_{{\text{2}}_{e}}-\;F_{{\text{2}}_{u}}\right))/\text{2})$$ (2)

Where F_1_ and F_2_ denote the first and second formant frequencies of vowels/a/,/i/,/u/, and/e/, respectively [25,26]. This qVSA method provided a more comprehensive measure of articulatory working space than the traditional triangular approach [27]. The calculated area values were compared across conditions, with and without the pseudo-palatal plate, to evaluate the device’s impact on vowel articulation precision.

### D. Jitter, shimmer and harmonics-to-noise ratio (HNR)

Local jitter is defined as the average absolute difference between consecutive F0 periods, normalized by the average period duration [28]. It is calculated using Equation (3).


$$local\;Jitter\;(\%)=\frac{\frac{\text{1}}{N-\text{1}}\sum_{i=\text{1}}^{N-\text{1}}{\;(T}_{i}-T_{i+\text{1}})}{\frac{\text{1}}{N}\sum_{i=\text{1}}^{N}T_{i}}$$ (3)

Where T_i_ represents the extracted F_0_ period lengths, and N is the total number of extracted F_0_ periods.

Shimmer measures cycle-to-cycle amplitude variation in the vocal signal and reflects the stability of vocal fold vibration intensity [29,30]. In this study, shimmer was calculated from sustained vowel segments following consonant articulation. Two shimmer metrics were computed: local shimmer (%) and shimmer in decibels (dB). Local shimmer was calculated using Equation (4).


$$local\;Shimmer\;(\%)=\frac{\frac{\text{1}}{N-\text{1}}\sum_{i=\text{1}}^{N-\text{1}}{\;(A}_{i}-A_{i+\text{1}})}{\frac{\text{1}}{N}\sum_{i=\text{1}}^{N}A_{i}}$$ (4)

Where A_i_ denotes the extracted peak-to-peak amplitude values, and *N* is the number of extracted F_0_ periods.

HNR represents the proportion of periodic (harmonic) energy to aperiodic (noise) energy in a voice signal and serves as an index of voice quality [31]. Lower HNR values are indicative of hoarseness or dysphonia, while higher values reflect clearer phonation. In this study, HNR was derived from the same vowel segments using Praat’s autocorrelation-based algorithm and expressed in decibels. HNR was calculated using Equation (5).


$$HNR\;(dB)=\text{1}\text{0}\times \text{log }(\frac{Harmonics\;energy}{Noise\;energy})$$ (5)

High HNR values are characteristic of healthy voices, whereas values below 7 dB are commonly associated with pathological vocal conditions [32].

## Experimental methods

### A. Palatal plate fabrication and testing environment

**Fig 2:**
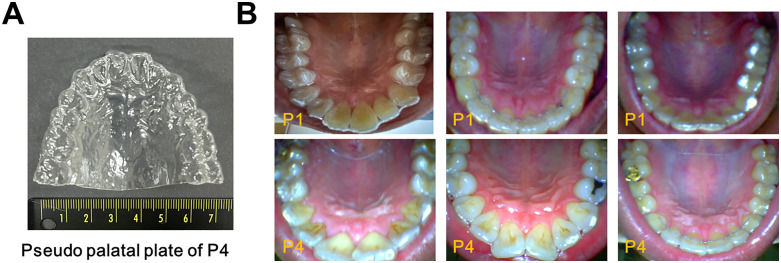
(A) Fabricated palatal plate and (B) Experimental environment for data acquisition and photographs of the oral cavity wearing a fabricated palatal plate.

The palatal plate used in this study was a custom-fabricated oral appliance designed to fit securely against the user’s hard palate. Its fabrication process resembled that of dental aligners such as Invisalign. Initially, an impression of the participant’s upper dental arch was obtained to accurately capture palatal morphology. Dental plaster was then poured into the impression to produce a rigid mold replicating the subject’s maxillary structure. Once the plaster mold had fully set, it was separated from the impression. A thin sheet of biocompatible plastic was thermoformed over the hardened cast to create the palatal plate. The final appliance had a uniform thickness between 80 and 100 µm, minimizing interference with natural articulation. As shown in **Fig 2(A)**, the fabricated plate was mounted onto the participant’s maxillary teeth and palate. To promote familiarity and reduce initial discomfort, each participant was instructed to wear the plate for a minimum of 30 minutes before performing speech tasks.

As illustrated in **Fig 2(B)**, each participant was instructed to wear a palatal plate and articulate the designated target consonant, which was visually presented on a computer screen. Each target syllable was repeated three times per condition. The experimental procedure was controlled using E-Prime software (version 3.0; Psychology Software Tools, USA), which managed the timing and presentation of visual stimuli. Each trial lasted 5 seconds and followed a fixed sequence: (1) fixation cross (500 ms), (2) blank screen (250 ms), (3) target syllable presentation (1,000 ms), (4) blank screen (250 ms), and (5) pronunciation phase (3,000 ms). Target syllables were presented in randomized order to minimize potential learning effects and anticipatory bias. Prior to the main experiment, participants rested for 10 minutes and were acclimated to both the palatal plate and speech tasks. All experimental procedures were conducted under the supervision of a licensed speech-language pathologist. Testing took place in a quiet, air-conditioned laboratory maintained at approximately 26.5 °C, with ambient noise levels controlled between 30 and 35 dB SPL. Speech recordings were captured using a condenser microphone (PURER, Joytron, Republic of Korea) positioned 30 cm from the participant’s mouth. Recordings were sampled at 22 kHz with a bitrate of 352.8 kbps. Each target syllable was produced five times by each participant, both before and after wearing the palatal plate. Acoustic feature analysis, including formants, jitter, shimmer, and HNR, was performed using Praat software (ver. 6.3.09; Boersma & Weenink, University of Amsterdam, Netherlands), a widely used tool in speech science. MFCCs were extracted, and corresponding correlation coefficients were computed using a Python program (ver. 3.10.12; Python Software Foundation, USA) developed with the Librosa (ver. 0.10.1) and NumPy (ver. 1.24.3) libraries.

### B. Pronunciation materials

To evaluate the acoustic effects associated with wearing a palatal plate, participants were instructed to wear the device and articulate target syllables containing either consonants or vowels. **Table 1** lists the target consonants used in the experiment, categorized according to standard phonetic dimensions, including place of articulation and phonation type (plain, tense, aspirated) [33]. As shown in Table 1, a limited subset of consonants was intentionally selected to enable controlled comparisons across articulation types while minimizing phonetic and contextual variability. Because the pseudo-palatal plate is attached to the palatal region, the target consonants were chosen to involve tongue positions most likely to interact with the palate. Accordingly, consonants with minimal tongue–palate contact, such as nasals, rhotics, and lateral approximants, were excluded from the present study. The target consonants included the alveolar/t/, post-alveolar/tʃ/, and velar/k/. For consonant analysis, correlation coefficients derived from MFCCs and the F2 slope were calculated across phonation types and articulation positions. **Table 2** summarizes the target vowels, which cover a range of tongue positions, with emphasis on back and low positions. Acoustic evaluation of vowel production included measurements of the first three formant frequencies (F1–F3), local jitter (%), local shimmer (%), HNR (dB), and qVSA.

**Table 1:** Target consonants of the speech materials used in the experiment.

Articulation position/Sounds	Target consonant	Observation
alveolar/plain (PA)	/t/	Correlation coefficients based on the MFCC, ΔMFCC, and ΔΔMFCC F2 slope
alveolar/tense (TA)	/t’/	
alveolar/aspirated (AA)	/t^h^/	
alveolar-palatal/plain (PAp)	/tΣ/	
alveolar-palatal/tense (TAp)	/ tΣ’/	
alveolar-palatal/aspirated (AAp)	/ tΣ^h^/	
velar/plain (PV)	/k/	
velar/tense (TV)	/k’/	
velar/aspirated (AV)	/k^h^/	

MFCC, Mel-frequency cepstral coefficient; F2, second formant.

**Table 2:** Target vowels of speech materials used in the experiment.

Tongue position (Height/Backness)	Target vowels	Observation
Low/Mid-back	/a/	Formants 1–3, Jitter local (%), Shimmer local (%), HNR (dB), qVSA
Low/Front	/e/	
High/Front	/i/	
Mid-high/Back	/o/	
High/Back	/u/	

HNR, harmonics-to-noise ratio; qVSA, quadrilateral vowel space area.

### C. Participants

All procedures involving human participants were reviewed and approved by the Institutional Review Board of Daegu University (IRB No. 1040621-201907-HR-061-02). The study adhered to applicable ethical guidelines and regulations and the Declaration of Helsinki. The study prospectively recruited healthy adult volunteers between October 7, 2021, and October 6, 2022; written informed consent was obtained from all participants. No minors or other vulnerable populations were included. Six healthy adults (four males, two females; mean age: 33.9 years; age range: 25–40 years) participated. All were native Korean speakers with intermediate English proficiency. None reported a history of speech or neurological disorders. Cognitive, auditory, and visual functions were confirmed to be within normal limits based on the Korean version of the Mini-Mental State Examination (K-MMSE). Participants didn’t receive compensation. No personally identifiable information is included in this manuscript.

### D. Statistical analysis

Statistical analyses were performed using R Studio (version 4.4.2; Posit, Boston, MA, USA). In the consonant-based experiment, the F2 slope data were analyzed to examine the effects of articulation manner (plain, tense, aspirated). A nonparametric Friedman test was used to compare F2 slopes across articulation types, followed by post-hoc pairwise comparisons with Bonferroni correction. To assess the impact of wearing the palatal plate on the F2 slope across different articulation positions, the Wilcoxon signed-rank test was applied. In the vowel-based experiment, linear regression analysis was conducted to evaluate changes in formant frequencies (F1–F3) before and after plate application. Furthermore, the Wilcoxon signed-rank test was used to compare acoustic features, including F1, F2, F3, fundamental frequency (f₀), jitter, shimmer, and HNR, between conditions with and without the palatal plate.

## Results

### A. Correlation coefficients based on MFCCs

**Fig 3:**
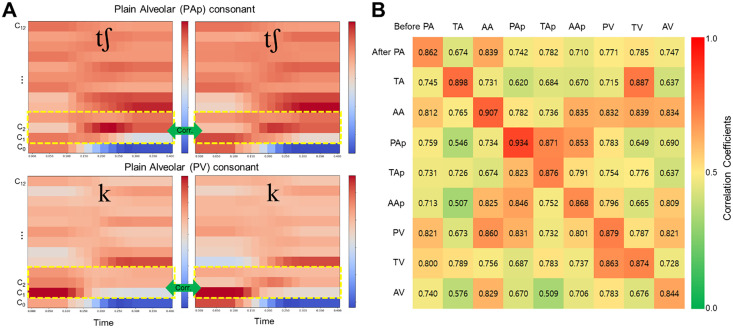
(A) Heatmaps visualizing the MFCC coefficient values for the consonants/tʃ/ and/k/, before (left) and after (right) wearing the palatal plate. The horizontal axis represents time, and the vertical axis represents MFCC coefficient indices. The yellow dashed box highlights the specific coefficient range used in the correlation analysis. (B) Correlation coefficient matrix comparing MFCC patterns across different articulation conditions. Red indicates higher correlations (greater acoustic similarity), while green suggests lower correlations.

**Fig 3** presents the MFCC and corresponding correlation results for target consonants before and after application of the pseudo-palatal plate. In **Fig 3(A)**, heatmaps illustrate the MFCCs for the consonants/tʃ/ and/k/, with pre-plate data shown on the left and post-plate data on the right. MFCC comparisons were based on the first three coefficients (C_1_–C_3_), and were applied uniformly across MFCC, ΔMFCC, and ΔΔMFCC representations. From these feature sets, a total of nine coefficients were extracted and used to calculate Pearson correlation coefficients for each articulation condition. Mean correlation values are shown in **Fig 3(B)**. The highest similarity was observed in the same articulation position under the tense condition, with mean correlation coefficients ranging from 0.844 to 0.934 across pre- and post-plate conditions. In contrast, comparisons across different articulation positions showed lower correlation values, typically ranging from 0.5 to 0.8. However, notable exceptions were observed. For example, a high correlation (0.887) was found between tense consonants/tʃ/ (after alveolar) and/k/ (before velar), despite differing articulation positions. Similarly, moderate correlations were observed when articulation position remained constant but the tense condition varied, such as 0.863 (after TV vs. before PV) and 0.871 (after PAp vs. before TAp). Overall, the strongest correlations consistently occurred under conditions where both articulation position and consonant tenseness were matched.

### B. F2 slope

**Fig 4:**
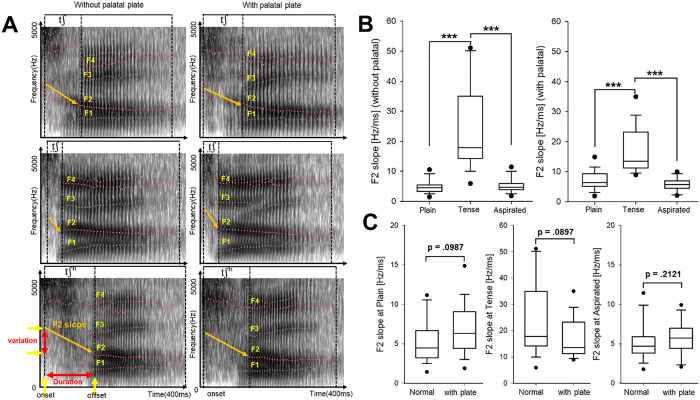
(A) Spectrograms visualizing the formant structures and second formant (F2) slope for the consonants/tʃ/,/τ−Σ tΣ’/, and/ tΣ^h^/, before (left) and after (right) wearing the palatal plate. (B) Box plots comparing F2 slope comparisons across three articulation types: plain, tense, and aspirated. A nonparametric Friedman’s test revealed a significant main effect (p < .001), and post-hoc pairwise comparisons were conducted using the Bonferroni correction. (C) Box plots comparing F2 slope between normal and plate conditions for each articulation manner. No statistically significant difference was found based on the Wilcoxon signed-rank test (p > .05 for all comparisons).

**Fig 4** illustrates the acoustic analysis of the F2 slope before and after wearing the palatal plate. In **Fig 4(A)**, spectrograms depict the trajectories of formant frequencies (F1–F4) during the articulation of alveolar-post consonants/tʃ/,/tʃ/, and/tʃʰ/, with pre-plate data shown on the left and post-plate data on the right. The F2 slope, calculated as the rate of frequency change over time (ΔF2/Δt), is visually represented by yellow lines and red arrows. Notably, tense consonants consistently exhibited the steepest F2 transitions, regardless of the plate-wearing condition.

**Fig 4(B)** presents box plots comparing F2 slope values across three articulation types: plain, tense, and aspirated, with and without the palatal plate. Significant differences were found among the articulation types in both conditions (p < 0.001; Friedman’s test with Bonferroni correction), with the tense consonants showing consistently higher F2 slope values. In contrast, plain and aspirated sounds did not differ significantly from each other. To further explore the influence of the palatal plate, **Fig 4(C)** presents F2 slope comparisons between normal and plate-wearing conditions across each articulation type. Although none of the differences reached statistical significance (Wilcoxon signed-rank test, p > .05 for all comparisons), slight increases in F2 slope were noted for the plain (p = 0.0987) and tense (p = 0.0897) conditions. No appreciable effect was observed for the aspirated condition (p = 0.2121). These findings suggest that while the presence of the palatal plate does not significantly alter the F2 slope, it may marginally enhance articulatory precision in high-tension environments, particularly for tense consonants.

Nevertheless, the results presented in **Fig 4** suggest that consonant articulation remains largely resilient to interference from the palatal plate.

### C. Formants (F1 to F3)

**Fig 5:**
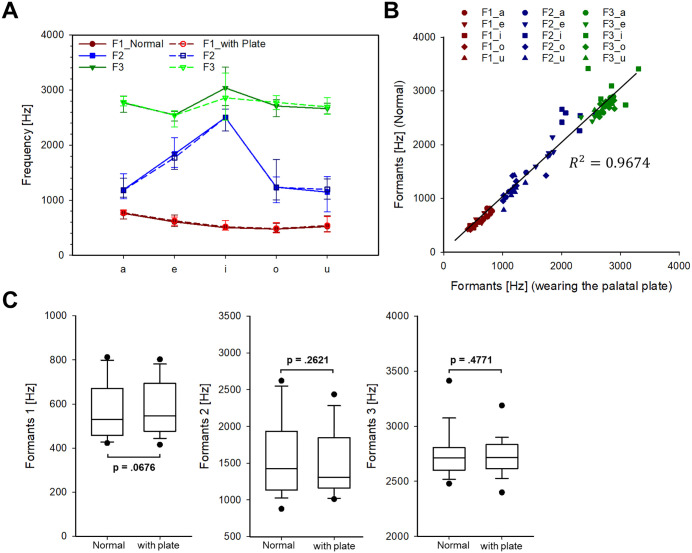
Comparison results of vowel formant frequencies (F1, F2, and F3) for normal and wearing the palatal plate. (A) Line plots of average formant values for five vowels (/a/,/e/,/i/,/o/,/u/) under two conditions: normal test (solid lines) and wearing the plate (dashed lines). (B) Linear regression analysis showing the correlation between formant values under normal and plate-wearing conditions; and (C) box plots comparing the distribution of F1, F2, and F3 between the two conditions.

As shown in Table 2, an acoustic comparison of vowel production was conducted to examine changes in formant frequencies before and after wearing the palatal plate. **Fig 5(A)** shows the average formant values (F1, F2, F3) for five vowels (/a/,/e/,/i/,/o/, and/u/), produced under both conditions. Across all vowels, formant patterns remained generally consistent between the normal (solid lines) and plate-wearing (dashed lines) conditions. However, a little deviation was observed for the vowel/i/, which involves a high-front tongue position; specifically, F3 differed by approximately 180–200 Hz between conditions. To assess the consistency of the formant structures, **Fig 5(B)** presents a linear regression analysis comparing formant values across conditions. The analysis yielded a high correlation (R² = 0.9668), indicating that the overall formant characteristics were preserved despite the presence of the palatal plate. Furthermore, **Fig 5(C)** provides box plots comparing the distribution of each formant (F1, F2, and F3) between the two conditions. No statistically significant differences were found for any formant (Wilcoxon signed-rank test, p > 0.05 for all comparisons). Collectively, these findings suggest that vowel articulation is largely resilient to interference from the palatal plate. However, minor acoustic deviations may occur in specific vowel contexts that require more extreme tongue positions.

### D. qVSA

**Fig 6:**
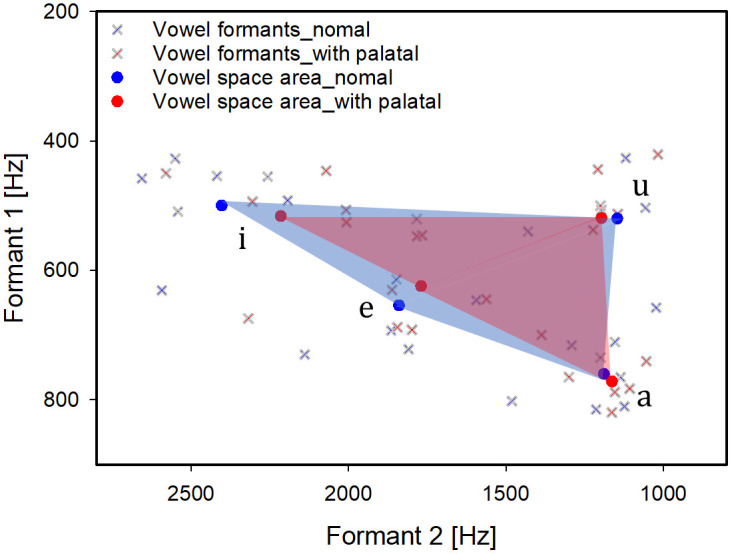
Comparison results of the quadrilateral vowel space area (qVSA) before and after wearing the palatal plate. The qVSA was calculated using the F1–F2 coordinates of the corner vowels/i/,/e/,/a/, and/u/. Blue and red shaded regions indicate the vowel space under normal (without plate) and wearing (with plate) conditions, respectively.

To assess articulatory working space during vowel production, the qVSA was calculated using the first and second formant frequencies (F1 and F2) of four corner vowels:/i/,/e/,/a/, and/u/. This analysis aimed to determine whether the palatal plate constrained tongue movement or reduced the range of vowel articulation. **Fig 6** displays the qVSA results under both conditions. The blue-shaded region represents the vowel space in the normal condition, while the red-shaded region corresponds to the plate-wearing condition. Although shifts in F1 and F2 values were observed, particularly for the high-front vowel/i/ and the low-front vowel/e/, the overall configuration of the vowel quadrilateral remained relatively stable. The average qVSA was 182,672.2 Hz² in the normal condition and 138,664.4 Hz² with the palatal plate, indicating an approximate 24.1% reduction in articulatory working space. While this reduction is relatively substantial, it did not result in perceptual confusion or syllable misidentification. Nevertheless, the findings suggest that the palatal plate may restrict tongue mobility, potentially due to altered intraoral volume or tactile interference. Overall, vowel articulation appears largely preserved, though the palatal plate may exert a measurable influence on articulatory range by limiting tongue displacement during vowel production.

### E. Fundamental frequency (f₀), jitter, shimmer, and HNR

**Fig 7:**
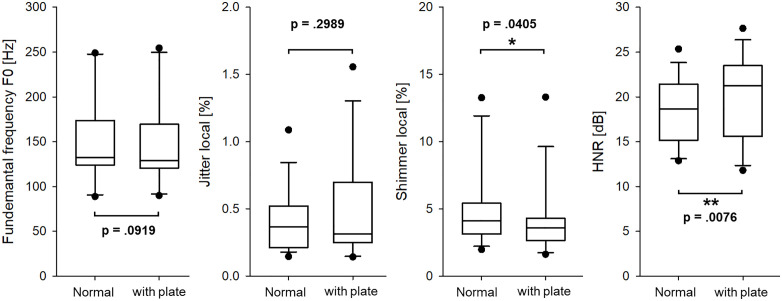
Comparison of acoustic parameters during vowel production before and after wearing the palatal plate.

In the vowel production task, additional acoustic parameters were analyzed to assess the impact of the palatal plate on vocal stability and intensity. These parameters included F₀, local jitter (%), local shimmer (%), and HNR. As shown in **Fig 7**, no statistically significant differences were found in F₀ (p = 0.0919) or jitter (p = 0.2989), indicating that the palatal plate did not affect the F₀ or frequency perturbation during vowel articulation. However, shimmer (p = 0.0405) and HNR (p = 0.0076) showed statistically significant changes, suggesting that the plate may influence the amplitude characteristics of vocal fold vibration. Specifically, the reduction in shimmer reflects decreased amplitude instability, while increases in HNR suggest enhanced vocal periodicity under the sensor-wearing condition. These findings imply that the palatal plate may affect glottal airflow or the vocal effort required during speech production. In particular, the subtle reduction in shimmer points to possible interference with airflow amplitude regulation, potentially due to increased intraoral resistance or altered resonance properties. Overall, while frequency-related features such as F₀ and jitter remained stable, amplitude-based acoustic indices appeared more sensitive to the presence of the palatal plate. Participant-level summaries of all key acoustic measures are provided in S1–S3 Tables to facilitate direct inspection of inter-individual variability.

## Discussion

Most prior research on intraoral devices has emphasized physical articulatory measurements using EPG, focusing primarily on contact location and timing. In contrast, the present study offers a novel perspective by examining the acoustic outcomes of speech, which more directly capture the perceptual effects of articulatory interference. The minimal acoustic deviations observed align with previous findings, indicating that thin, biocompatible palatal plates can be engineered to minimize disruption during articulation.

### A. Limitations and implications

Several limitations of the present study warrant consideration. First, shimmer and HNR are susceptible to variation due to recording conditions and individual phonatory differences, which may have introduced uncontrolled noise into the dataset. In vowel production, slight but statistically significant changes in shimmer and HNR were observed following insertion of the palatal plate. Specifically, local shimmer decreased by an average of 0.72%, while HNR increased by approximately 2.5 dB. Shimmer quantifies cycle-to-cycle amplitude perturbation in the voice signal, whereas HNR reflects the relative contribution of harmonic energy to aperiodic noise. Although shimmer and HNR are often discussed in relation to voice quality, the present study does not directly assess glottal-source characteristics such as vocal fold vibration or airflow. Rather, the device altered the intraoral acoustic environment, thereby affecting the speech signal as captured by the microphone. The rigid palatal surface may have reduced variability in oral resonance patterns or supraglottal acoustic conditions, which could indirectly influence amplitude-related acoustic measures such as shimmer and HNR. Thus, while the observed changes were modest, they suggest that intraoral devices may subtly influence acoustic parameters traditionally attributed to phonatory stability, primarily by modifying the vocal tract’s filtering characteristics. Accordingly, the observed changes in shimmer and HNR are interpreted here as indirect acoustic correlates rather than direct evidence of altered glottal source behavior. Future studies incorporating dedicated glottal-source analyses, such as glottal flow estimation or excitation-based feature extraction, would be valuable for elucidating the physiological mechanisms underlying the observed acoustic patterns. Prior work on glottal-source feature analysis provides a methodological foundation for such investigations [34,35].

In this study, the average qVSA was 182,672.2 Hz² under normal conditions and 138,664.4 Hz² with the palatal plate, representing an approximate 24% reduction in articulatory working space. Prior research has reported qVSA values ranging from 204,000–291,000 Hz² for males and 313,000–456,000 Hz² for females aged 16 and older [36], indicating that the present values obtained in this study are comparatively lower. Notably, the measured F1 frequency for the vowel/i/ ranged from 500 to 550 Hz, diverging from the typical 300–400 Hz range reported in previous studies. This discrepancy likely reflects the limited sample size, which constrains the generalizability of these findings to broader populations or individuals with speech and neurological impairments. Future research should therefore include larger and more diverse participant cohorts, particularly clinical populations, to more fully assess the acoustic impact of palatal plates in contexts where articulatory sensitivity is heightened.

### B. Tongue-interface devices and potential acoustic impact

Compared with a thin palatal plate designed to minimize interference (thermoformed biocompatible sheet, < 100 µm), tongue-interface devices that occupy larger palatal volume or introduce stiffer surfaces are expected to exert greater effects on oral resonance and tongue kinematics. In our data, the thin plate produced modest but measurable acoustic changes (e.g., a reduction in qVSA with largely preserved formant structure, along with small, directionally consistent decreases in shimmer and increases in HNR) that are more plausibly attributed to filter-level effects within the vocal tract rather than direct alterations in glottal source stability.

Our experimental findings also have implications for the design of emerging tongue-interface technologies, which commonly rely on custom, user-fitted sensor systems. MouthPad [37] is a custom, palate-mounted, pressure-sensitive touchpad fabricated from dental resin; public specifications describe an overall device thickness of ~1 mm, a mass of ~7.5 g, and an approximate 80 × 50 × 30 mm envelope, seated on the roof of the mouth and communicating via Bluetooth. Such added mass/volume and localized stiffness are likely to reduce adequate oral-cavity volume and constrain anterior–posterior tongue excursions, particularly for high front and high back vowels. Another device is the Inductive Tongue Computer Interface [38]. The inductive TCI embeds multiple inductive sensors in a dental retainer; an activation unit couples to the tongue, enabling invisible intraoral control. Also, this system emphasizes usability and explicitly considers the potential impact on speaking, drinking, and eating in end-users. Given its retainer-style construction (thicker and mechanically stiffer than a < 100 µm plate), we anticipate resonance-volume reduction and articulation constraint. A further line of work involves tongue pressure sensors [39]. These systems place screen-printed pressure sensors on a plastic substrate against the palate, with conditioning/transmission electronics for wireless acquisition. Designs target minimal invasiveness (no intraoral cabling) but still add a substrate and rigid elements that can locally stiffen the palatal surface. Consequently, qVSA may decrease, and extreme vowels may exhibit mild F1–F2 drift.

Although intraoral sensors and tongue-interface devices continue to diversify for convenience and accessibility, their potential speech-related effects must be considered when deployed intraorally. In particular, device thickness, palatal coverage, stiffness, and mass, as well as user adaptation time, jointly determine the extent of resonance alteration and articulatory constraint. The present findings with a palatal plate offer acoustic evaluation methods (e.g., qVSA and dynamic indices such as F2 slope together with jitter/shimmer/HNR) that can be directly applied when assessing newer tongue interfaces with larger physical footprints.

## Conclusion

This study investigated the acoustic effects of a pseudo-palatal plate on speech production, examining consonant and vowel articulation via MFCC-based correlations, F2-slope dynamics, and formant trajectories. Except for shimmer, HNR, and qVSA, the presence of the plate produced only minor acoustic deviations. Notably, tense consonants retain robust formant-transition patterns irrespective of plate use. The average qVSA decreased by 24.1% with the palatal plate and showed a trend toward reduction; shimmer decreased slightly (−0.72%), and HNR increased (+2.5 dB). Meanwhile, the overall formant structure and consonant articulation were preserved mainly, consistent with a filter-level influence of the plate rather than a change in glottal source stability. Overall, the acoustic impact of a thin plate appears limited. Future work should increase sample size, include clinical populations, and systematically vary plate thickness, palatal coverage, and stiffness to establish design guidelines that minimize acoustic interference. Additionally, longitudinal assessment of adaptive speech behaviors and user comfort may offer deeper insights for optimizing intraoral device design.

## Supporting information

S1 Table Participant-level F2 slope (Hz/ms) across three articulation types before and after wearing the pseudo-palatal plate. (PDF)

S2 Table Participant-level formant frequencies (Hz) and quadrilateral vowel space area (Hz²) before and after wearing the pseudo-palatal plate. (PDF)

S3 Table Participant-level fundamental frequency (F0), local jitter, local shimmer, and harmonics-to-noise ratio (HNR) before and after wearing the pseudo-palatal plate. (PDF)
