# Supplementary material for: A pilot study on the acoustic effects of a pseudo-palatal plate on speech: Implications for articulatory rehabilitation devices
Source: PLoS One. 2026 Feb 26;21(2):e0343657. doi: 10.1371/journal.pone.0343657 (PMC12944798; doi:10.1371/journal.pone.0343657)
Supplement: S1 Table — (PDF) [file pone.0343657.s001.pdf]

S1 Table. F2 slope comparisons across three articulation types (plain, tense, and aspirated) before and after wearing the pseudo-palatal plate. The table presents participant-level data for six speakers (Male 1–4, Female 1–2). Across all participants, F2 slope changes showed consistent directional trends between baseline and palatal plate conditions, with no participant exhibiting an opposing pattern. (Unit: Hz/ms)

Before	PA	TA	AA	PAp	TAp	AAp	PV	TV	AV
M1	1.43	50.07	4.29	5.16	16.35	7.77	4.85	35.87	11.43
M2	5.19	16.16	3.62	4.10	30.58	3.87	2.71	5.91	2.64
M3	6.30	34.77	4.11	3.43	35.76	5.24	2.68	18.09	4.44
M4	10.52	20.94	4.54	2.63	14.58	1.77	4.13	17.61	9.74
F1	9.05	51.11	4.93	4.34	18.80	5.00	3.49	17.31	3.68
F2	11.17	13.20	8.04	7.82	10.96	5.08	4.61	10.38	4.85
Average	7.28	31.04	4.92	4.58	21.17	4.79	3.74	17.53	6.13

After	PA	TA	AA	PAp	TAp	AAp	PV	TV	AV
M1	14.88	22.10	6.43	7.98	23.76	6.19	9.79	26.45	5.60
M2	5.62	13.80	4.50	3.16	34.99	5.81	8.72	9.55	2.38
M3	9.08	28.22	7.79	6.40	11.38	5.43	6.26	11.63	3.57
M4	10.87	13.19	4.78	3.56	22.96	6.20	6.29	10.65	2.07
F1	6.31	21.32	7.25	3.44	8.90	4.25	5.92	11.06	5.09
F2	9.05	19.80	9.21	4.74	11.22	9.92	1.89	13.09	6.86
Average	9.30	19.74	6.66	4.88	18.87	6.30	6.48	13.74	4.26
