# Supplementary material for: A pilot study on the acoustic effects of a pseudo-palatal plate on speech: Implications for articulatory rehabilitation devices
Source: PLoS One. 2026 Feb 26;21(2):e0343657. doi: 10.1371/journal.pone.0343657 (PMC12944798; doi:10.1371/journal.pone.0343657)
Supplement: S3 Table — (PDF) [file pone.0343657.s003.pdf]

S3 Table. Participant-level fundamental frequency (F0), local jitter, local shimmer, and harmonics-to-noise ratio (HNR) measured before and after wearing the pseudo-palatal plate conditions. Participant-level comparisons indicated generally consistent trends across speakers, with no extreme outliers disproportionately influencing the median changes.

Before	F0 (Hz)	Jitter (%)	Shimmer (%)	HNR (dB)
M1	127.76	0.32	2.79	19.03
M2	91.62	0.87	11.53	13.92
M3	127.25	0.31	3.90	21.98
M4	140.54	0.19	3.88	18.58
F1	247.08	0.42	2.89	18.41
F2	205.34	0.29	3.87	19.24
Median	134.15	0.31	3.88	18.80
After	F0 (Hz)	Jitter (%)	Shimmer (%)	HNR (dB)
M1	124.87	0.27	3.35	20.71
M2	91.30	1.19	10.60	14.01
M3	127.29	0.30	3.51	23.22
M4	127.81	0.24	3.39	20.87
F1	250.61	0.80	3.11	16.51
F2	202.36	0.20	2.40	24.45
Median	127.55	0.28	3.37	20.79
