# Supplementary material for: A pilot study on the acoustic effects of a pseudo-palatal plate on speech: Implications for articulatory rehabilitation devices
Source: PLoS One. 2026 Feb 26;21(2):e0343657. doi: 10.1371/journal.pone.0343657 (PMC12944798; doi:10.1371/journal.pone.0343657)
Supplement: S2 Table — (PDF) [file pone.0343657.s002.pdf]

S2 Table. Participant-level formant frequencies and quadrilateral vowel space area (qVSA) measured before and after wearing the pseudo-palatal plate conditions. Although individual variability was observed, including an increase in qVSA in one participant (M2), no single participant disproportionately contributed to the observed group-level effect. (Unit: Hz for formant frequencies; Hz^2 for qVSA and ΔVSA)

Before	F1_a	F1_e	F1_i	F1_u	F2_a	F2_e	F2_i	F2_u	qVSA (Hz^2)	
M1	810	614	492	427	1124	1848	2593	784	306885	
M2	658	521	631	503	1023	1784	2542	1057	22790	
M3	711	646	509	507	1154	1595	2418	1197	128067	
M4	815	722	454	716	1214	1810	2257	1291	96708	
F2	765	693	455	427	1136	1865	2656	1119	318084	
F1	802	730	458	540	1481	2139	2550	1430	223503	
Average									182673	
After	F1_a	F1_e	F1_i	F1_u	F2_a	F2_e	F2_i	F2_u	qVSA (Hz^2)	ΔqVSA (After-Before)
M1	793	557	426	410	1107	1783	2071	1017	211352	-95533
M2	745	546	704	513	1054	1766	2318	1353	29276	+6486
M3	798	645	526	537	1154	1562	2007	1223	91884	-36183
M4	745	692	484	698	1205	1898	2205	1256	80601	-16107
F2	824	630	507	444	1164	1962	2008	1208	198375	-119709
F1	765	688	450	502	1299	1844	2581	1201	220499	-3005
Average									138664	-44008
